# Optical coherence tomography-measured retinal nerve fiber layer thickness values compensated with a multivariate model and discrimination between stable and progressing glaucoma suspects

**DOI:** 10.1007/s00417-021-05329-3

**Published:** 2021-08-05

**Authors:** Hemma Resch, Florian Schwarzhans, Florian Frommlet, Anton Hommer, Philipp Fuchs, Clemens Vass

**Affiliations:** 1 Department of Ophthalmology & Optometry, Medical University Vienna, General Hospital, Waehringer Guertel 18-20, A-1090 Vienna, Austria; 2 Section for Medical Information Management and Imaging, Center for Medical Statistics Informatics and Intelligent Systems, Medical University Vienna, Vienna, Austria; 3 Section for Medical Statistics, Center for Medical Statistics Informatics and Intelligent Systems, Medical University Vienna, Vienna, Austria; 4 Hommer Ophthalmology Institute, Albertgasse 39, Vienna, Austria

**Keywords:** Optic nerve, Glaucoma, Imaging, HRT/CSLT, OCT

## Abstract

**Purpose:**

Our previously introduced multivariate model, compensating for intersubject variability, was applied to circumpapillary retinal nerve fiber layer (RNFL) values measured with optical coherence tomography in glaucoma suspects with or without prior progressive optic disc (OD) change in a series of confocal scanning laser tomography (CSLT, HRT III) measurements.

**Methods:**

In this prospective study, OD change during CSLT follow-up was determined with strict, moderate, and liberal criteria of the topographic change analysis (TCA). Model compensation (MC) as well as age compensation (AC) was applied to RNFL sectors (RNFLMC vs. RNFLAC). Diagnostic performance of RNFLMC vs. RNFLAC was tested with an area under the receiver operating characteristic (AUROC) and was compared between methods.

**Results:**

Forty-two glaucoma suspects were included. Patients without prior progressive OD change during the CSLT follow-up (= stable) had thicker RNFL thickness values in most areas and for all progression criteria. RNFLMC AUROC for the global RNFL (0.719) and the inferior quadrant (0.711) performed significantly better compared with RNFLAC AUROC (0.594 and 0.631) to discriminate between stable and progressive glaucoma suspects as defined by the moderate criteria of CSLT progression analysis (*p* = 0.028; *p* = 0.024).

**Conclusion:**

MC showed a slight but significant improvement in detection of subjects with prior progressive OD change among a group of glaucoma suspects, when compared to AC, which is the compensation method commonly used during OCT data evaluation in daily routine. Further studies are warranted to validate the present results.


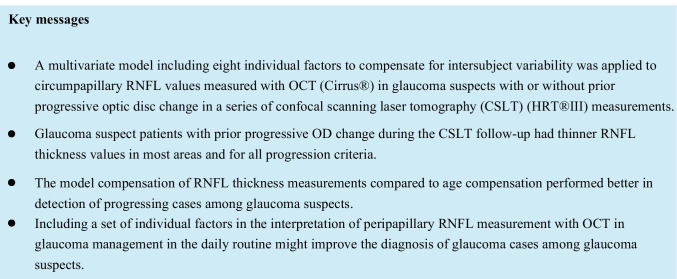


## Introduction

Glaucoma is a progressive optic neuropathy characterized by the irreversible loss of retinal ganglion cells and their axons, leading to visual field defects [1]. An accurate and early diagnosis of this chronic disease is crucial, as no cure is established.

Currently, early diagnosis of glaucoma, besides functional tests, is performed based on imaging techniques such as optical coherence tomography (OCT) [2]. This is done by measuring the circumpapillary retinal nerve fiber layer (RNFL) thickness and optic disc (OD) parameters and comparing the measurement results with a normative database. Despite a high reproducibility [3, 4] of RNFL thickness measurements, intersubject variance [5] due to the influence of numerous individual factors hampers the interpretation and use of imaging techniques, especially in the early diagnosis of glaucoma. Individual biomarkers, such as sex, age, and ethnicity [6–9], are typically taken into account by the normative databases. However, other factors are not, including optic disc and fovea parameters, retinal vessel position, axial length, and refractive error, although they show an association with circumpapillary RNFL thickness and distribution [10–19].

In a previous publication, which is in line with previous research, we could show a significant correlation of retinal blood vessel position and peripapillary RNFL thickness distribution [20–22]. To enable a more comprehensive analysis of this factor, we recently have developed the parameter of circumpapillary retinal vessel density (RVD)—a function dependent on thickness and distribution of the major circumpapillary retinal blood vessels [23]. We have shown that interindividual variance may be reduced, on average, by 11% and up to 20% when taking RVD into account.

In a further study [24], this parameter, together with seven other anatomical features (corresponding to OD and fovea descriptors, as well as age and refractive error), were included in a multivariate model. The sample used to develop the multivariate model consisted of 101 healthy subjects and the independent sample used to validate the model consisted of a different set of 101 healthy subjects. The model achieved a reduction of the coefficient of variation of 18% on average (up to 29%), when tested in the independent validation sample. This work provides strong evidence of the major impact that anatomical factors, especially the distribution of the retinal vessels, have on the RNFL thickness profile in healthy volunteers. The reduction of the coefficient of variance demonstrates that intersubject variance in the RNFL thickness profile of healthy subjects can be decreased, which may improve the imaging support of glaucoma diagnosis.

In the present study, we have included glaucoma suspects, an interesting patient group, as the differentiation between normal and suspect-looking optic discs in early glaucoma is very much complicated especially due to interindividual differences. Our previously introduced model, compensating for intersubject variability, was applied to circumpapillary RNFL values measured with OCT (Cirrus®) in glaucoma suspects with or without prior progressive OD change in a series of at least 4 confocal scanning laser tomography (CSLT) (HRT®III) measurements. The aim of the present study was to investigate whether glaucoma suspect patients with or without prior OD change are behaving differently in RNFL measurement with OCT and if reducing interindividual variability of RNFL measurements through using a multivariate compensation method might improve detection of glaucoma among glaucoma suspects.

## Materials and methods

### Subjects

This prospective study was performed in collaboration with the Department of Ophthalmology and Optometry and the Section for Medical Statistics, Center for Medical Statistics Informatics and Intelligent Systems, Medical University Vienna, Vienna, Austria, for data analysis.

### Inclusion and exclusion criteria

Forty-two glaucoma suspects were included. They were recruited from an ophthalmology institute, where follow-up examinations with the CSLT (HRT) during their glaucoma routine checks were done at least once a year prior to this study.

Inclusion criteria for glaucoma suspects were suspect appearance of the OD of at least one eye (either large excavation > 0.5, or asymmetry of excavation > 0.2, or localized rim loss, or failure of ISNT rule, or baring of circumlinear vessels) and reproducible normal visual fields (VF) in standard automated perimetry (SAP). Progressive OD change during a series of 3 of at least 4 consecutive CSLT follow-up measurements was determined with strict, moderate, and liberal criteria of the topographic change analysis (TCA) [25].

Patients with or without prior progressive OD change were divided into 2 groups, progressive or stable. This was done for all three TCA criteria.

We excluded all subjects with any evidence of other ocular pathology, history of ocular trauma or intraocular surgery, ocular inflammation or infection within the last 3 months, astigmatism more than + 2.0 diopters, and ametropia of more than ± 5.0 diopters. In addition to these criteria, only optic disc examinations acquired with Fourier Domain OCT (FD-OCT, Cirrus® Carl Zeiss Meditec Inc.) with a quality index higher than 5 were included (quality index was ranging from 0 to 10), as image quality may have an impact on RNFL thickness measurements [26]. Moreover, scans with movement artifacts within the measurement circle were excluded.

### Experimental paradigm

Initially, a complete ophthalmological examination was performed, including medical history, best-corrected Snellen visual acuity, slit-lamp examination, fundoscopy, measurement of IOP by Goldmann applanation tonometry, and VF examination.

Subjects eligible for participating in the study according to the inclusion/exclusion criteria were included. If both eyes were includable, one eye was selected randomly.

The study was performed at the Department of Ophthalmology and Optometry of the Medical University of Vienna.

### Methods

The included glaucoma suspect patients had CSLT follow-up measurements (at least 4 measurements) during their glaucoma routine checks at an ophthalmology institute. OD morphology with HRT III (Heidelberg Engineering GmbH, Heidelberg, Germany; software version 1.7) was measured without pupil dilation. In brief, a 3-dimensional topographic image consisting of 384 × 384 × 16 up to 384 × 384 × 64 voxels was constructed from multiple focal planes axially along the optic nerve head. To visualize the disc borders, the HRT images were inspected using the 3D image display. To define the contour line, six or more points were positioned at the inner margin of the scleral ring. This was done by one trained technician. Once the contour line was drawn, the software automatically calculated all the optic disc parameters. The reference plane is defined at 50 µm posterior to the mean retinal height between 350 and 356 degrees along the contour line. The area above the reference is defined as the rim and below as the cup. The standard protocol and the extended parameter table were then exported for statistical analysis.

OD change during the CSLT follow-up was determined with strict, moderate, and liberal criteria of the TCA [25]. The TCA takes into account the size and depth of the largest contiguous area or cluster of significant change within the optic disc border. According to that, patients were divided into a stable and progressive glaucoma suspect group.

On the study day, an automated visual field testing was performed with the Humphrey field analyzer II (program 30–2). Visual field eligibility criteria were less than 33% false-positive responses, less than 33% false-negative responses, and less than 33% fixation losses. Subjects showing either pathologic glaucoma hemifield test or a cluster of 3 points in pattern deviation plot significant at 0.5% not located at the border of the visual field, were excluded. As well, Cirrus® -SD-OCT (Carl Zeiss Meditec, Dublin, CA, USA) measurements centered in the macula (Macular Cube 200 × 200) and centered on OD were performed by one technician, using the optic disc cube 200 × 200. With this program, a data cube of 6-mm^2^, which acquires as a series of 200 horizontal scan lines, each composed of 200 A-scans was recorded. Measurements of the OD parameters were automatically generated by a Carl Zeiss Meditec OD analysis algorithm developed for Cirrus® -SD-OCT (software version 10.0.0.14618) without the interaction of the technician [27]. The RNFL was measured at a 3.4-mm-diameter circle around the OD. For both parameters, the software calculates the average, superior, nasal, inferior, and temporal quadrant values. Model compensation (MC) as well as age compensation (AC) was applied to the global, quadrants, and clock hour sectors of these RNFL thickness values.

### Statistical analysis

Statistical analysis was performed using the SPSS® software package (Version 26.0.0, SPSS Inc., USA) and R Version (3.6.0) [28]. Multivariate model compensation (MC), (for details see our previous manuscript) [24] as well as age compensation (AC) of RNFL (RNFLMC vs. RNFLAC) was applied to the global, quadrants, and clock hour sectors (CHR) of the peripapillary RNFL thickness values, for both patient groups, stable and progressive. Parameters included in the multivariate model were age, spherical refractive error, retinal vessel density, optic disc area, orientation, and ratio (quotient between major and minor axis), as well as disc-fovea distance and angle. These parameters are known to correlate with peripapillary RNFL distribution. To compensate for the effect of the above eight parameters on RNFL thickness, we used the multivariate model to calculate for each subject a model-compensated RNFL thickness (RNFLMC). For age compensation, we used the data of the instrument’s normative database. Differences between groups regarding RNFL (RNFLMC and RNFLAC) and age were assessed using the Mann–Whitney *U* tests and Student’s *t* test. *P* values are provided as an explorative tool and were not corrected for multiple testing. *P* values smaller than 0.05 indicate the difference between groups.

The diagnostic performance of RNFLMC vs RNFLAC vs originally measured RNFL was tested with the area under the receiver operating characteristic (AUROC) and compared between methods using DeLong’s tests computed with the R package pROC [29].

## Results

From the 54 glaucoma suspects screened, 12 had to be excluded due to insufficient image quality and/or retinal pathologies (for example vitreomacular traction) or visual field defects. A total of 42 glaucoma suspects with or without prior progressive OD change in a series of at least 4 CSLT (HRT®III measurements) were included. The mean follow-up time of measurements with HRT was 104.4 months. For statistical analysis, the last 3 measurements next to the study day were used. Subjects’ baseline demographics are provided in Table 1.

**Table 1:** Demographic data. Patient characteristics, *n* = 42 glaucoma suspects. *SD* standard deviation, *IOP* intraocular pressure, *MD* mean deviation, *C/D* cup/disc ratio

	*n* = 42 glaucoma suspects
Female:male	23:19
Mean age ± SD in years	62.0 ± 11.4
Mean ± SD of total CSLT follow-up in months	104.4 ± 39.2
Mean number ± SD of CSLT examinations	9 ± 3
IOP on the study day (mean ± SD) (mmHg)	16 ± 3.57
Visual field MD (mean ± SD) (dB)	− 0.1 ± 1.25
C/D ratio (mean ± SD)	0.7 ± 0.07
Refractive error, spheric equivalent (mean ± SD) (diopters)	− 1.04 ± 2.02
Number of patients with glaucoma therapy	10

OD change during the CSLT follow-up was determined with strict, moderate, and liberal criteria of the TCA. According to that, patients were divided into a stable and a progressive patient group for each of the three criteria. The number of glaucoma suspects with prior progressive optic disc change during CSLT follow-up was 38, 29, and 16 for liberal, moderate, and strict criteria of the TCA, respectively. The number of stable glaucoma suspects was 4 for the liberal, 13 for the moderate, and 26 for the strict criterion.

The application of the strict criterion of TCA leads to fewer numbers of progressive glaucoma suspect patients due to its conservative definition of requiring a significant cluster of ≥ 2% of the disc area and a depth change of ≥ 100 µm. In contrast, the liberal criterion required a significant cluster of ≥ 0.5% of the disc area and a depth change of ≥ 20 µm and the moderate criterion a significant cluster of ≥ 1% of the disc area, and a depth change of ≥ 50 µm.

MC as well as AC of RNFL was applied to the global peripapillary RNFL thickness values measured with OCT, as well as to the quadrants thereof. For each instance, the differences in RNFL thickness and also in age between the two glaucoma suspect groups (stable versus progressive) were assessed with *t-*tests (Table 2).

**Table 2:** Median values of peripapillary retinal nerve fiber layer thickness values (RNFL) in µm (and interquartile range) using original, model compensated (MC), and age compensated (AC) values for the two groups of glaucoma suspect patients, with (= progressive) or without (= stable) prior progressive optic disc change during the CSLT follow-up

TCA	Progressive	Stable	*P* values
Liberal criteria (*n*)	38	4	
Age	62.0 (± 11.7)	58.8 (± 6.1)	0.561
RNFL global	85.5 (80.5–91.4)	**98.5** (92.6–101.8)	0.008*
Temporal quadrant	62.0 (56.3–70.1)	62.5 (54.3–70.1)	0.932
Superior quadrant	104.6 (92.7–115.6)	**132.8** (116.8–144.6)	0.007*
Nasal quadrant	67,7 (64.4–78.5)	68.7 (64.0–79.4)	0.830
Inferior quadrant	106.7 (96.2–116.2)	**126.0** (116.5–135.9)	0.015*
RNFL global_MC	85.7 (80.9–88.7)	**97.0 (94.0–99.0)**	0.004*
Temporal quadrant_MC	61.5 (56.2–66.6)	**63.6** (55.4–75.3)	0.607
Superior quadrant_MC	103.6 (93.5–115.5)	**121.4** (118.1–134.7)	0.004*
Nasal quadrant_MC	70.4 (65.0–74.6)	**73.1** (68.8–76.2)	0.346
Inferior quadrant_MC	105.4 (96.3–113.9)	**126.6** (118.8–135.0)	0.005*
RNFL global_AC	87.7 (84.0–94.5)	**100.7** (94.2–105.1)	0.016*
Temporal quadrant_AC	64.3 (57.2–71.4)	63.8 (55.1–71.7)	1.000
Superior quadrant_AC	108.3 (97.0–118.7)	**135.7** (118.4–149.2)	0.008*
Nasal quadrant_AC	70.1 (65.6–79.5)	70.0 (65.6–80.2)	0.898
Inferior quadrant_AC	109.0 (101.9–122.1)	**130.0** (120.5–139.5)	0.015*
Moderate criteria (*n*)	29	13	
Age	65.0 (± 10.8)	55.3 (± 8.7)	0.003*
RNFL global	85.4 (80.3–90.1)	**91.5** (84.5–96.7)	0.062*
Temporal quadrant	**65.8** (56.6–70.6)	58.3 (54.4–65.1)	0.178
Superior quadrant	103.1 (91.9–113.2)	**115.4** (105.8–124.7)	0.027*
Nasal quadrant	**67.9** (64.7–75.8)	66.8 (63.5–81.5)	0.881
Inferior quadrant	105.4 (95.5–115.1)	**114.1** (105.5–127.9)	0.055
RNFL global_MC	84.0 (79.8–88.2)	**89.4** (85.7–96.0)	0.025*
Temporal quadrant_MC	**63.6** (58.0–68.2)	57.4 (55.3–65.2)	0.178
Superior quadrant_MC	103.3 (88.5–113.5)	**115.4** (101.7–118.6)	0.066*
Nasal quadrant_MC	69.1 (65.0–74.4)	**72.0** (68.5–75.5)	0.391
Inferior quadrant_MC	104.9 (93.8–113.1)	**114.3** (103.6–126.6)	0.031*
RNFL global_AC	87.8 (83.9–94.3)	**92.6** (85.2–99.2)	0.334
Temporal quadrant_AC	**67.9** (58.8–72.2)	58.8 (55.3–66.3)	0.084*
Superior quadrant_AC	108.1 (96.7–117.0)	**114.3** (106.1–127.3)	0.131
Nasal quadrant_AC	**70.1** (66.4–77.8)	68.7 (64.4–82.3)	0.838
Inferior quadrant_AC	109.2 (100.8–122.1)	**118.3** (106.1–129.7)	0.178
Strict criteria (*n*)	16	26	
Age	66.6 (± 10.1)	58.7 (± 11.1)	0.02*
RNFL global	86.0 (76.5–88.9)	**87.3** (83.1–95.3)	0.133
Temporal quadrant	**67.3** (56.9–72.3)	59.8 (55.3–67.0)	0.120
Superior quadrant	102.4 (89.5–115.8)	**107.4 (97.7–119.8)**	0.178
Nasal quadrant	65.9 (62.8–71.5)	**71.4** (65.0–80.2)	0.062*
Inferior quadrant	101.6 (93.3–111.0)	**113.1** (103.4–124.1)	0.024*
RNFL global_MC	85.3 (78.8–88.2)	**87.1** (83.5–94.3)	0.087*
Temporal quadrant_MC	**64.8** (60.0–68.5)	58.4 (55.7–66.2)	0.178
Superior quadrant_MC	98.2 (92.8–114.1)	**106.5** (99.3–117.3)	0.204
Nasal quadrant_MC	67.3 (65.0–72.6)	**72.3** (66.3–77.2)	0.062*
Inferior quadrant_MC	101.1 (91.2–111.2)	**112.6** (101.5–107.4)	0.034*
RNFL global_AC	86.9 (81.7–93.9)	**88.1** (85.4–98.0)	0.254
Temporal quadrant_AC	**70.1** (59.5–73.6)	60.4 (55.8–68.9)	0.059*
Superior quadrant_AC	108.5 (94.1–120.5)	**110.3** (101.1–121.4)	0.484
Nasal quadrant_AC	67.9 (65.0–74.2)	**72.4** (65.6–81.8)	0.147
Inferior quadrant_AC	105.8 (99.4–116.0)	**115.6** (104.9–125.8)	0.074*

Group status was determined with liberal, moderate, and strict criteria of the topographic change analysis (TCA). Differences between groups were assessed using Student’s *t* test. *P* values are provided as an explorative tool and were not corrected for multiple testing. **Bold** = thicker RNFL values (> 1 µm, statistical significance not required), *P values smaller than 0.1 indicate some difference between groups

Glaucoma suspect patients without prior progressive OD change during the CSLT follow-up (= stable) were significantly younger and had thicker RNFL thickness values in most areas and for all progression criteria. The only exception was the temporal quadrant for the moderate and strict criteria for originally measured RNFL as well as RNFLMC and RNFLAC, where the stable patients had thinner RNFL values, although generally not statistically significant. For the liberal criterion, stable glaucoma suspects (this were only 4 patients) had statistically significant larger RNFL thickness values globally and in the superior and inferior quadrant, originally measured but also after applying MC and AC. For the strict criterion, progressive glaucoma suspects had statistically significant thinner RNFL thickness values in the inferior quadrant, again for all three methods, originally measured and after applying MC and AC.

Diagnostic performance of RNFLMC vs RNFLAC was quantified with area under the receiver operating characteristic (AUROC) and compared between methods using DeLong’s test (Table 3). Due to the significant age differences between the stable and the progressive groups, we do not report in detail on the AUROC as obtained by the originally measured RNFL.

**Table 3:** Comparison of diagnostic performance of multivariate model compensation (MC) as well as age compensation (AC) of retinal nerve fiber layer (RNFL) thickness parameters using area under the receiver operating characteristics (AUROC) for liberal, moderate, and strict criteria of the topographic change analysis in glaucoma suspect patients

	AC AUROC	MC AUROC	*P* values
Liberal criteria			
RNFL global	0.868	**0.947**	0.107
Temporal quadrant	0.500	0.579	0.805
Superior quadrant	**0.908**	**0.947**	0.460
Nasal quadrant	0.520	0.645	0.450
Inferior quadrant	0.875	0.928	0.234
Moderate criteria			
RNFL global	0.594	**0.719**	0.028*
Temporal quadrant	0.332	0.371	0.436
Superior quadrant	**0.647**	0.679	0.449
Nasal quadrant	0.480	0.584	0.594
Inferior quadrant	0.631	0.711	0.024*
Strict criteria			
RNFL global	0.606	0.659	0.354
Temporal quadrant	0.325	0.375	0.277
Superior quadrant	0.565	0.618	0.398
Nasal quadrant	0.635	0.673	0.656
Inferior quadrant	**0.666**	**0.697**	0.401

**Bold** = best performing parameters in terms of AUROC regarding MC or AC (cutoff ≥ 0.01) *P values smaller than 0.05 indicate the difference between groups

For glaucoma suspects, in general, liberal criteria of HRT progression performed better concerning AUROC than moderate or strict criteria (Table 3), regardless of the compensation method. But the interpretation that the liberal criterion is better regarding diagnostic separation is speculative, as a small sample size in the stable group (*n* = 4) impedes significant results.

**Fig. 1:**
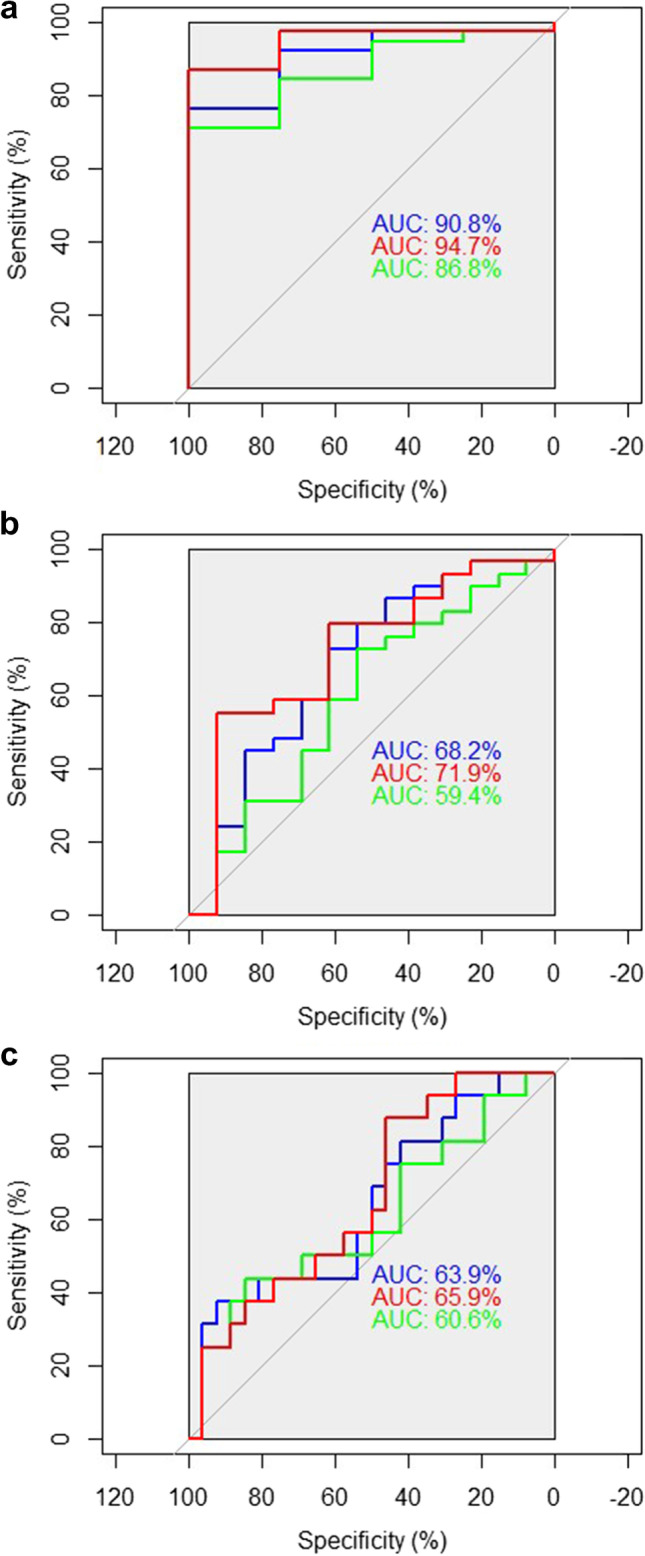
**a** AUC global RNFL thickness parameters, *liberal criteria*. Blue, RNFL_Average (original); red, RNFL_Average (model compensated, MC_AUROC); green, RNFL_Average (age compensated, AC_AUROC). **b** AUC global RNFL thickness parameters, *moderate* criteria. Blue, RNFL_Average (original); red, RNFL_Average (model compensated, MC_AUROC); green, RNFL_Average (age compensated, AC_AUROC). **c** AUC global RNFL thickness parameters, *strict criteria*. Blue, RNFL_Average (original); red, RNFL_Average (model compensated, MC_AUROC); green, RNFL_Average (age compensated, AC_AUROC)

MC AUROC of global RNFL and of the inferior quadrant performed statistically significantly better than AC AUROC, for the moderate criterion (see Fig. 1a–c for the AUROCs of the RNFL global).

In general, original RNFL and MC AUROC performed better than AC AUROC for the moderate progression criterion. For the strict criterion, these differences were less pronounced.

AUROC for originally measured RNFL performed statistically significantly better than AC AUROC for the superior, nasal, and inferior quadrant for moderate and strict criteria and for RNFL global for the moderate criterion. But taking into account the age distribution for stable and progredient glaucoma suspects, it became obvious that stable glaucoma suspects were statistically significantly younger than progredient ones by 10 or 8 years for the moderate and the strict criteria. This leads to bias in favor of the originally measured RNFL. In Fig. 1, the AUROCs for global RNFL thickness parameters are shown and the respective curves for the originally measured RNFL do not take into account the age differences, while both the AC and the MC do correct for age differences.

## Discussion

In this paper, we use our prior published multivariate model that facilitates a clinically meaningful reduction of the intersubject variance of peripapillary RNFL parameters, by compensating for variation of parameters correlating with RNFL thickness distribution. This model includes physiological parameters, automatically extracted from OCT fundus images such as retinal vessel density, OD shape, fovea location, and additionally age and refractive error [23, 24].

This model (MC) was applied on RNFL parameters measured with Cirrus OCT in patients with suspicion of glaucoma. This patient collective was divided into 2 groups, a group with or without prior progressive OD change in a series of at least 4 HRT®III measurements. Change during the CSLT follow-up was determined with strict, moderate, and liberal criteria of the TCA. MC as well age compensation (AC) of RNFL was applied to the global, quadrants, and clock hour sectors of RNFL thickness values. It is to be expected that the MC leads to a better diagnostic separation. For glaucoma suspect patients, trends are seen in the present paper of MC performing better in regards of AUROC in comparison to AC, for all progression criteria, with statistically significant differences for global RNFL and the inferior quadrant (a clinically relevant region for glaucoma damage) for the moderate criterion. The lack of significance for all other parameters might be on the one hand due to the small sample size, especially for stable patients according to the liberal criteria. On the other hand, the limitation of HRT as a method to detect glaucoma progression (OD change over time) might have had an influence.

There is no consensus regarding the ability of HRT to detect progression among the prior literature. One study showed that TCA parameters observed longitudinally can discriminate between progressing and healthy eyes [25]. However, another prospective study suggested that TCA progression criteria do not predict photographic evaluation or visual field progression [30]. Similarly, a retrospective study demonstrated that the stereometric parameters of the HRT did not have a high enough sensitivity and specificity to detect glaucomatous progression that was otherwise detected by serial stereoscopic OD photography evaluated by experienced masked observers [31]. There are certain factors that can influence measurement reliability. The HRT estimates the height location of surface pixels indirectly through the use of a reference plane, which is set at an arbitrarily determined depth below the outer limit of the RNFL in the papillomacular bundle. This reference plane may be subject to alteration due to progressing glaucomatous damage at this location. This in turn may affect changes in pixel height as assessed by TCA.

In the present paper, 10 patients were treated with eye pressure–lowering eye drops, only 1 patient needed therapy enhancement (from mono-therapy to a combination treatment), and no glaucoma surgery was performed during the HRT measurement period. An influence of treatment on the measurement results seems thus unlikely.

The better performance of the AUROCs for liberal criteria in glaucoma suspect patients when compared to the other two criteria might indicate that in our setting a large proportion of patients with progressive OD change according to liberal criteria truly have glaucoma whereas, for strict criteria, many of them are labeled as (false) negatives. On the other hand, only when progression was defined by the moderate criterion the inferior quadrant RNFL MC and global RNFL MC performed significantly better than AC.

In our data, AUROC for originally measured RNFL performed statistically significantly better than RNFL AC AUROC for the superior, nasal, and inferior quadrant for moderate and strict criteria. However, lack of age compensation introduces a relevant bias when using the originally measured RNFL values in our data, where progressive glaucoma suspects were older by 8 to 10 years compared to stable glaucoma suspects. Furthermore, it is the AC compensation method, which is commonly applied during OCT data evaluation in daily routine, whereas the originally measured RNFL values, without age correction or age-dependent normative values, are generally neither clinically nor scientifically used.

A sample bias might also have had an influence on the study results, as patients with newly developed visual field defects were excluded. Only patients with normal visual fields were recruited, but if they had reproducible scotomas on the study day, they were excluded from further study participation. This was the case in 10 glaucoma suspects, which might lead to an inclusion of only stable glaucoma suspects. For these 10 glaucoma suspects, we also executed the strict, moderate, and liberal criteria of the TCA. For the liberal criterion, all of these glaucoma suspects were labeled as progressive (10 out of 10). Regarding the moderate criterion, 6 out 10, and, for the strict criteria, 4 out of 10, glaucoma suspects were classified with progressive optic disc change. The interpretation of this analysis could be that with strict criteria of the HRT-TCA real glaucoma cases might be missed; and on the other hand, for the liberal criterion, healthy subjects might be labeled as glaucoma. CSLT is able to detect optic disc change over time, but its clinical impact regarding real glaucoma disease progression should be interpreted with caution.

In conclusion, we found some evidence that the reduction of interindividual variability of RNFL measurements through using a multivariate compensation method might improve the detection of glaucoma among glaucoma suspect patients. Further studies are warranted to validate the present results.

## Data Availability

On demand.

## Code availability (software application or custom code)

On demand.
